# Isolation of native plasma membrane H^+^‐ATPase (Pma1p) in both the active and basal activation states

**DOI:** 10.1002/2211-5463.12413

**Published:** 2018-03-25

**Authors:** Jesper Torbøl Pedersen, Tamara Kanashova, Gunnar Dittmar, Michael Palmgren

**Affiliations:** ^1^ Department of Plant and Environmental Sciences University of Copenhagen Frederiksberg Denmark; ^2^ Mass Spectrometry Core Unit Max Delbrück Center for Molecular Medicine Berlin Germany; ^3^ Proteome and Genome Research Laboratory Luxembourg Institute of Health Strassen Luxembourg; ^4^ Present address: Institute of Environmental Medicine (IMM) Karolinska Institutet Stockholm Sweden

**Keywords:** acetylated tubulin, autoinhibitory regulation, Pma1p, P‐type ATPase

## Abstract

The yeast plasma membrane H^+^‐ATPase Pma1p is a P‐type ATPase that energizes the yeast plasma membrane. Pma1p exists in two activation states: an autoinhibited basal state and an activated state. Here we show that functional and stable Pma1p can be purified in native form and reconstituted in artificial liposomes without altering its activation state. Acetylated tubulin has previously been reported to maintain Pma1p in the basal state but, as this protein was absent from the purified preparations, it cannot be an essential component of the autoinhibitory mechanism. Purification of and reconstitution of native Pma1p in both activation states opens up for a direct comparison of the transport properties of these states, which allowed us to confirm that the basal state has a low coupling ratio between ATP hydrolysis and protons pumped, whereas the activated state has a high coupling ratio. The ability to prepare native Pma1p in both activation states will facilitate further structural and biochemical studies examining the mechanism by which plasma membrane H^+^‐ATPases are autoinhibited.

**Abbreviations**
ACMA: 9‐amino‐6‐chloro‐2‐methoxyacridine
cymal‐7: 7‐cyclohexyl‐1‐heptyl‐β‐d‐maltoside
LDAO: n‐dodecyl‐N,N‐dimethylamine‐N‐oxide
OD_600_: optical density
OG: n‐octyl β‐d‐glucopyranoside
PM: plasma membrane

The ~100 kDa plasma membrane (PM) H^+^‐ATPase, which generates an electrochemical gradient that drives the transport of other solutes across the PM, is a major protein in fungal and plant PMs 1, 2, 3. The PM H^+^‐ATPase belongs to the P‐type ATPase family; all members of this family studied to date share the same overall fold and form a phosphorylated reaction cycle intermediate during transport (reviewed in 4, 5). Many P‐type ATPases contain autoinhibitory domains that regulate pumping activity, and such domains have been identified in the C termini of both plant and fungal PM H^+^‐ATPases 6, 7. The autoinhibitory C‐terminal domain maintains the pump in a basal state, which is characterized by a low affinity for ATP (*K*_m_ ≈ 2.5 mm) and an apparent low coupling ratio (if any) between ATP hydrolysis and protons pumped. Within minutes of sensing glucose (in the case of fungi; refs. 8, 9, 10) or blue light (in plants; ref. 11, 12, 13), residue(s) in the C terminus are phosphorylated and potential regulatory proteins are attracted. This forces the C‐terminal domain to release its constraint on the pump, allowing it to enter the activated state, with a high affinity for ATP (*K*_m_ ≈ 0.5 mm) and presumably tight coupling 14, 15, 16.

The genome of the yeast *Saccharomyces cerevisiae* encodes two PM H^+^‐ATPase isoforms, the essential and highly expressed Pma1p and the nonessential and weakly expressed Pma2p 17. Pma1p is the most studied fungal PM H^+^‐ATPase, and knowledge of the autoinhibitory regulation of fungal PM H^+^‐ATPases originates primarily from this pump. When a yeast cell senses glucose, a number of events trigger the full activation of the autoinhibited Pma1p. Glucose sensing induces phosphorylation of at least two residues in the C‐terminal region of Pma1p (Ser‐911 and Thr‐912) 9, 10 and changes the distribution of this protein in the PM, causing Pma1p oligomers to cluster in small areas 18. How the C‐terminal domain inhibits the catalytic function of the pump is unknown. Mutational studies have identified residues in the cytosolic core domains that, when altered, change the activation state of the protein. The C terminus is thought to interact with the cytosolic part of the core protein 19, 20, which might lead to an unstable phosphorylated intermediate that results in uncoupling of ATP hydrolysis from H^+^ transport 21. It has been reported that Pma1p interacts with acetylated tubulin, which may stabilize the pump in its basal state 22. A mechanism has also been proposed according to which glucose sensing leads to activation of a serine protease, that in turn causes hydrolysis of acetylated tubulin and its dissociation from the pump, thereby forcing Pma1p into the activated state 23.

A high‐resolution structure of the pump protein in the basal state would provide important clues into the mechanics of autoinhibition; however, such a structure is lacking. Solubilizing a membrane protein from its native environment without altering its conformational equilibrium is challenging, and both terminal truncations and the addition of affinity tags may affect the function of the protein. For example, it has previously been reported that it is impossible to solubilize the related plant PM H^+^‐ATPase without influencing the activation state of the protein 24, 25. The only available crystal structures of a PM H^+^‐ATPase are the 3.6 Å structure of a C‐terminally truncated and a 5.5 Å full‐length PM H^+^‐ATPase (AHA2) from the plant *Arabidopsis thaliana* 26. Both structures are in the activated state and do not reveal the localization of either of the terminal domains. Even though purification protocols for fungal PM H^+^‐ATPases were published more than 30 years ago 27, 28, 29, the only reported crystal structure of a fungal PM H^+^‐ATPase is a cryo‐electron microscopy structure of a *Neurospora crassa* PM H^+^‐ATPase, and the resolution of this structure (8 Å) was too low to locate the C‐terminal regulatory domain 30, 31.

In this study, we present a purification protocol for native *S. cerevisiae* Pma1p that does not employ affinity tags. We further show that the basal state of Pma1p can be isolated with high yield and purity without disrupting its autoinhibition using the detergent 7‐cyclohexyl‐1‐heptyl‐β‐d‐maltoside (cymal‐7) and the reactive dye Reactive Red 120. Furthermore, Pma1p can be stabilized with P‐type ATPase inhibitors in both the E1P and E2P conformation. This purification protocol lays the foundation for obtaining a high‐resolution structure of Pma1p in both the basal and activated state, and for advancing studies of single molecule transport by P‐type H^+^ pumps, which recently has become possible 32.

## Materials and methods

### Yeast strain and growth conditions

Strain YAK2 of *S. cerevisiae* (MAT, *ade2*–101, *leu2*Δ1, *his3*Δ200, *ura*3–52, *trp*1Δ63, *lys2*–801 *pma1*Δ::HIS3, *pma2*Δ::TRP1) was used, with yeast *PMA1* placed under its own promoter in a centromeric *LEU2* plasmid 33. The yeast was grown at 30 °C in minimal medium containing 2% glucose. The optical density at 600 nm (OD_600_) was determined at 1‐h intervals for 28 h, and cells used for protein purification were harvested after 24 h. Cells producing Pma1p in the activated and basal states were prepared as described previously 10.

### Isolation of plasma membranes and solubilization of Pma1p

PMs were isolated and loosely bound proteins removed by a chaotropic wash as described 21. The isolated and washed PMs were resuspended in a buffer containing 50 mm MES/KOH (pH 6.5), 20% glycerol, 1 mm EDTA, 1 mm dithiothreitol (DTT), 2 mm sodium molybdate, and 2 μg·mL^−1^ pepstatin A. The following detergents from the detergent screen DSOL‐MK, Anatrace, Maumee, OH, USA were tested for their ability to solubilize Pma1p from the PM: n‐octyl β‐d‐glucopyranoside (OG), ANAPOE^®^‐C_10_E_9_, C_12_E_8_ and C_13_E_8_, ANAPOE^®^‐X‐100 and X‐305, ANCERGENT^®^ 3‐10, CHAPSO, CYCLOFOS™‐7, 7‐cyclohexyl‐1‐pentyl‐β‐d‐maltoside, 7‐cyclohexyl‐1‐hexyl‐β‐d‐maltoside, 7‐cyclohexyl‐1‐heptyl‐β‐d‐maltoside (cymal‐7), FOS‐CHOLINE^®^‐ISO‐11, FOS‐MEA^®^‐10, PMAL™‐C8, n‐decyl‐a‐d‐maltopyranoside, FOS‐CHOLINE^®^‐12 and 16, hexaethylene glycol monooctyl ether (C_8_E_8_), and n‐dodecyl‐N,N‐dimethylamine‐N‐oxide (LDAO). Solubilization of Pma1p from the PM was tested in a 1 : 3 protein to detergent ratio for all detergents and from 1 : 1 to 1 : 5 for detergents that could solubilize Pma1p. PM and detergents were incubated in the resuspension buffer at 4 °C and 25 °C for 30 min with slow agitation, and nonsolubilized material was removed by ultracentrifugation for 1 h at 100 000 g at 4 °C.

### Reactive Red 120 purification

Reactive Red 120 beads from Sigma‐Aldrich^®^ (St. Louis, MO, USA) were equilibrated with 10 column volumes (CVs) of resuspension buffer with a 2 × critical micelle concentration of detergent (wash buffer). Solubilized Pma1p was incubated on a column for 1 h at 4 °C with slow agitation. Five milligrams of protein was used per milliliter of Reactive Red 120. The column was washed with 5 × CV wash buffer, and bound proteins were eluted first with 5 × CV wash buffer supplemented with 1 m KCl and then with 5 × CV wash buffer supplemented with 5 mm ADP. All fractions were collected and concentrated in an Amicon Pro^®^ affinity concentrator with 30‐kDa cutoff filters.

### Reconstitution into lecithin liposomes

The Pma1p in the activated and basal state was reconstituted into lecithin liposomes as described earlier 21 with the exception that only the detergents used in this study were employed.

### Pma1p stability

The Pma1p was incubated at 4 °C in a buffer containing 50 mm MES/KOH (pH 6.5), 20% glycerol, 50 mm KCl, 1 mm DTT, and 1 mm MgCl_2_. The buffer was supplemented with either 1 mm AlF_x_ (1 mm AlCl_2_ and 4 mm NaF), 1 mm ADP‐AlF_x_ (1 mm ADP, 1 mm AlCl_2_, and 4 mm NaF), or nothing. The protein was incubated for between 4 h and 3 weeks, and protein degradation was analyzed using immunoblot detection with a Pma1p‐specific antibody (sc‐33735 antibody from Santa Cruz Biotechnology, Dallas, TX, USA).

### ATPase activity measurements

The ATPase activity was determined using the Baginski assay 34. The assay was carried out at 30 °C in buffer containing 20 mm MES/KOH at pH 5.9, 10 mm MgSO_4_, 0–6 mm ATP, 50 mm KNO_3_, 5 mm NaN_3_, 0.44 mg·mL^−1^ phosphoenolpyruvate, 4 μg·μL^−1^ pyruvate kinase, and 3.5 mm Na_2_MoO_4_. The assay buffers were equilibrated to 30 °C, and the assay was started by adding 150 ng protein to 60 μL ATPase buffer in microtiter plates as described 25. All experiments were performed in triplicate with ± SE.

### Proton transport and coupling ratio

Proton transport into vesicles was measured using fluorescence quenching of 9‐amino‐6‐chloro‐2‐methoxyacridine (ACMA). Only the neutral form of ACMA can pass membranes freely, and therefore, the dye cannot leave a vesicle again following its protonation in the vesicle lumen. It is not known how ACMA mediates fluorescence quenching; however, as ACMA is an acridine derivative, the mechanism is likely similar to that described for acridine orange 35. Acridine orange dimerizes when its concentration increases locally and, as the dimer has a different absorbance and fluorescence spectrum compared to the monomer, the initial quenching of fluorescence is linear relative to the amount of H^+^ accumulating in vesicles 35. Assuming that a similar mechanism operates for ACMA, fluorescence changes during the H^+^ pumping reaction reflect the formation of protonated dimeric dye complexes inside vesicles, but do not directly report either the H^+^ concentration or ΔpH.

H^+^ pumping and ATP hydrolysis assays were carried out simultaneously in the same sample 36. The H^+^‐ATPase assay was conducted in 96‐well microtiter plates in a buffer containing 10 mm MES/KOH (pH 6.5), 50 mm K_2_SO_4_, 5 mm ATP, 2 mm phosphoenolpyruvate, 30 μg·mL^−1^ pyruvate kinase, 25 μg·mL^−1^ lactate dehydrogenase, 0.5 μg·mL^−1^ valinomycin, 0.25 mm NADH, 2 μm ACMA, and various protein concentrations in a final volume of 150 μL. In this assay, ATP hydrolysis is coupled to NADH oxidation. Pma1p in both activation states was reconstituted using the detergent OG as described above. The assay was started by adding MgSO_4_ to a final concentration of 8 mm, and fluorescence quenching was monitored at excitation/emission wavelengths of 412/480 nm (ACMA) or 350/440 nm (NADH) to minimize spectral overlap between the probes 36. The pH gradient was collapsed by adding 3 μg nigericin. Linear regression was used to compare ATP hydrolysis and proton uptake rates as follows: The linear regression was calculated in the linear area of both the drop in NADH and ACMA fluorescence. The concentration of purified Pma1p in the basal state was increased until the velocity constant of NADH fluorescence equaled the constant for 2.5 μg purified Pma1p in the activated state. The coupling ratio for Pma1p in the basal state was estimated using the difference between the velocity constant of ACMA fluorescence of the two activation states assuming that Pma1p in the activated state transports one proton per ATP hydrolyzed (the theoretical maximum) 14. All experiments were carried out in triplicate with ± SE.

### Mass spectrometric analysis

Fractions enriched in Pma1p were analyzed using tandem LC‐MS/MS analysis. The proteins were converted to peptides by applying endopeptidases Lys‐C (Wako Pure Chemical Industries, Wako, Japan) and trypsin (Promega, Madison, WI, USA). The peptides were purified using C18 stage‐tips (3 m) according to Rappsilber *et al*. 37 and separated on an in‐house‐packed analytical reverse‐phase column (0.075 mm × 200 mm, 3 μm Reprosil C18, Dr. Maisch GmbH) using a 4–76% acetonitrile gradient over 1 h on a Proxeon Easy‐nLC system (Proxeon Biosystems, Roskilde, Denmark). The samples were measured on a Q‐Exactive mass spectrometer (Thermo Scientific, Waltham, MA, USA). MS acquisition was performed at a resolution of 70 000 in the scan range from 300 to 1700 m/z. Dynamic exclusion was set to 20 s and the normalized collision energy to 26 eV. The mass window for precursor ion selection was set to 2.0 m/z. The recorded spectra were analyzed using the maxquant software package version 1.2.2.5 38 by matching the data to the UniProt *S. cerevisiae* database (version of 06 May, 2012) with a false discovery rate of 1% for proteins and peptides, allowing a maximum of two missed cleavages. Variable modifications were set to ‘oxidation of methionines’ and ‘acetylation of N termini’, whereas fixed modifications were set to ‘carbamidomethylation of cysteines’. All other parameters were set to the default values of the software.

### Protein concentration determination

The protein concentrations were determined using Bradford reagent and bovine serum albumin as a standard 39.

### SDS/PAGE and immunoblot

Sodium dodecyl sulfate polyacrylamide gel (SDS/PAGE) electrophoresis and immunoblotting were performed according to standard techniques. For Pma1p detection, the sc‐33735 antibody (Santa Cruz Biotechnology) was used, and for acetylated tubulin, 6‐11B‐1 (Sigma‐Aldrich) 40.

## Results

### Expression of Pma1p in *Saccharomyces cerevisiae*

The PM H^+^‐ATPase proteins encoded by the two genes in *S. cerevisiae* (*PMA1* and *PMA2*) show 89% sequence identity at the amino acid sequence level; however, *PMA1* is expressed at a much higher level than *PMA2* 41. To ensure a homogenous preparation with only one PM H^+^‐ATPase isoform, we used a yeast strain in which *PMA2* has been deleted (Yak2) 33. To provoke starvation and thereby keep Pma1p in the basal state, the yeast was harvested after 24 h in the stationary phase (Fig. S1). An average of 16 g of cells was harvested per liter of medium.

### Purification of Pma1p

It was previously reported that detergent solubilization partially activates the plant counterpart of Pma1p 24, 25. To test whether this also applies to Pma1p, we tested the ability of 20 different detergents to solubilize Pma1p in the basal state from the isolated PMs. For the detergents that could solubilize Pma1p, we determined the apparent activity after relipidation to evaluate the activation state. Pma1p in the activated state solubilized with octyl glucoside (OG) was used as a control, as OG has previously been shown to solubilize Pma1p in a functional state 14. Four detergents were able to solubilize Pma1p from the PM, and after relipidation, ATPase activity could be restored in Pma1p solubilized with OG, cymal‐7, and LDAO, but not C_12_E_8_ (Table 1). Using an increase in apparent affinity for ATP (decrease in *K*_m_) as a measure of protein activation, neither OG, cymal‐7 nor LDAO activated Pma1p when solubilized and relipidated, indicating that the overall conformation is not altered by the process. The optimal protein to detergent ratio was found to be 1 : 3 for OG and 1 : 4 for LDAO and cymal‐7.

**Table 1:** Kinetic parameters of Pma1p in the basal and activated state and the effect of detergent treatment. The apparent activity was determined as specific activity (μmol P_i_/mg protein/min) (*n* = 3 biological replicates; ± SE). n.a., not analyzed

Protein state	Detergent	Protein:Detergent ratio	mg protein·L^−1^ media	*K*_m_ [ATP]	*V*_max_
Basal state					
Plasma membrane	–	–	1.2	2.4 ± 0.7	4.0 ± 0.5
Solubilized	OG	1 : 3	0.9	2.3 ± 0.6	1.9 ± 0.2
Solubilized	cymal‐7	1 : 4	0.8	2.5 ± 0.4	3.5 ± 0.1
Solubilized	C_12_E_8_	1 : 4	0.5	n.a.	n.a.
Solubilized	LDAO	1 : 4	1.0	2.1 ± 0.7	0.9 ± 0.3
Reactive Red 120	cymal‐7	1 : 3	0.6	2.3 ± 0.3	4.1 ± 0.2
Activated state					
Plasma membrane	–	–	1.2	0.6 ± 0.1	11.7 ± 0.3
Solubilized	OG	1 : 3	0.9	0.6 ± 0.1	5.5 ± 0.2
Reactive Red 120	cymal‐7	1 : 3	1.0	0.4 ± 0.2	12.5 ± 0.9

**Figure 1:**
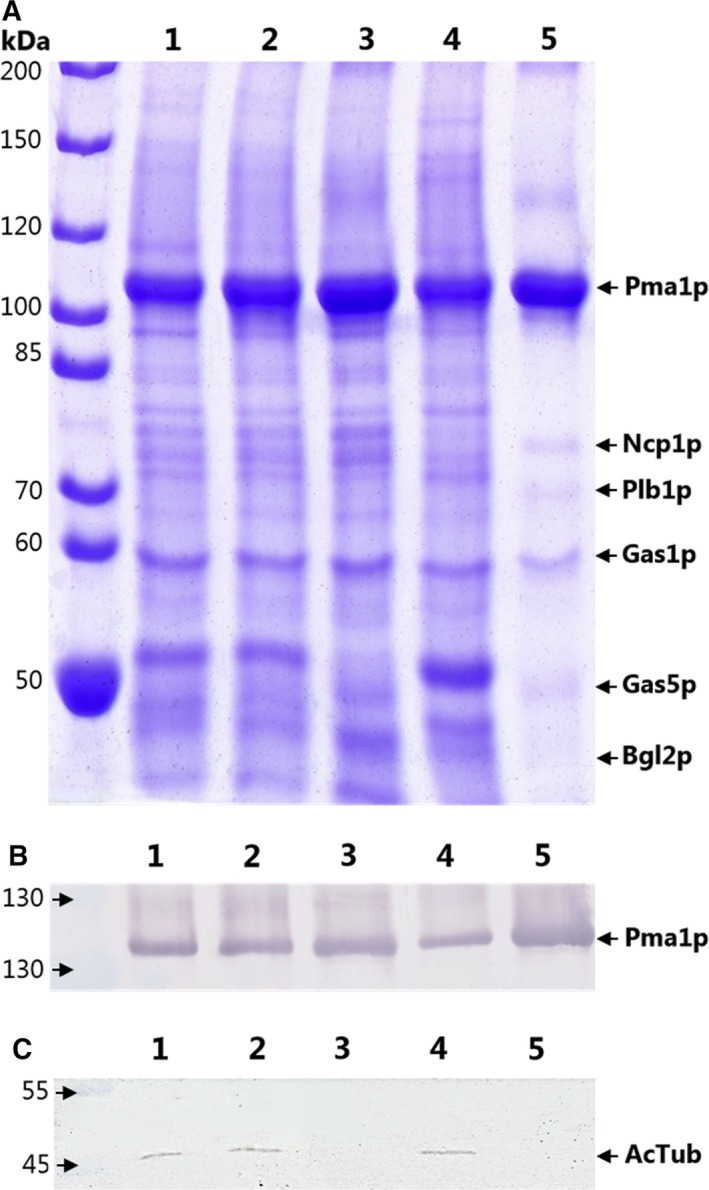
Purity of Pma1p in the basal state. The purity of Pma1p at different purification steps analyzed using (A) SDS/PAGE; 10% and (B) immunoblot with specific Pma1p antibodies. Ten micrograms of protein was used for the SDS/PAGE and 3 μg for the immunoblot. (C) Immunoblot of acetylated tubulin. Lane 1, crude plasma membrane fraction; lane 2, crude PM stripped with a KCl wash; lane 3, cymal‐7‐solubilized Pma1p; lane 4, nonsolubilized material; lane 5, Reactive Red 120‐purified Pma1p. LC‐MS/MS‐identified contaminating proteins are marked in lane 5.

*Arabidopsis thaliana* AHA2 was previously purified without loss in activity using a combination of affinity chromatography facilitated by a hexahistidine tag and anion‐exchange chromatography 26, 42. However, reports of functional purification tags and successful column chromatography purification of fungal PM H^+^‐ATPase are sparse, and only size exclusion chromatography has been shown to be somewhat useful for purifying the solubilized protein 43, 44. The P‐type sarco/endoplasmic reticulum (SERCA) Ca^2+^‐ATPase was previously shown to bind to the reactive dye Reactive Red 120, and this binding is readily disrupted after applying adenosine di‐ or triphosphate 45. We therefore tested whether Reactive Red 120 had similar binding properties for Pma1p. Proteins bound to the column could be eluted with a high concentration of KCl, whereas neither ADP nor ATP eluted any protein. However, contrary to our expectation, only a small proportion of cymal‐7‐solubilized Pma1p was retained by the column, in contrast to most of the contaminating proteins that were retained (Fig. 1).

The initial flow through enriched in Pma1p was concentrated and relipidated, and the apparent activity was tested. Pma1p in the basal state showed the same kinetic properties as the autoinhibited pump in membranes (Table 1). The drop in *V*_max_ in both solubilized and enriched protein compared to Pma1p in the PM is likely caused by a fraction of Pma1p facing inward in the lipid vesicles and therefore being shielded from ATP.

### Absence of acetylated tubulin in purified Pma1p preparation

Even though the purity of Pma1p has been improved with this protocol, some contaminating proteins were still present (Fig. 1A). As it has been reported that acetylated tubulin is essential for keeping Pma1p in the basal state 22, 23, we tested whether one of the contaminating proteins was acetylated tubulin. The anti‐acetylated tubulin antibody only decorated a band in the PM fractions and in the nonsolubilized material, but was absent from the purified fraction (Fig. 1C). We further analyzed the protein composition of the purified Pma1p preparation using quantitative liquid chromatography–tandem mass spectrometry (LC‐MS/MS). The 10 major contaminants were primarily cell wall proteins (Table 2, Fig. 1A); interestingly, we did not identify any tubulins (for full list of proteins, see Table S1).

**Table 2:** Proteins present in the Pma1p preparation. Proteins were identified by LC‐MS/MS. Polypeptides with an intensity of above 2.5 × 10^9^ are shown. For the full list, see Table S1

Protein ID	Description		Intensity × 10^9^
YGL008C	Pma1p	99.6 kDa	778
YOR270C	Vph1p (vacuolar ATPase, VO domain)	95.5 kDa	2.5
YHR042W	Ncp1p (NADP‐cytochrome reductase)	76.7 kDa	2.9
YMR008C	Plb1p (phospholipase B)	71.6 kDa	20
YMR307W	Gas1p (cell wall protein)	59.5 kDa	6.7
YKL103C	Ape1p (vacuolar aminopeptidase)	57.0 kDa	4.2
YOL030W	Gas5p (cell wall protein)	51.8 kDa	13
YBR078W	Ecm33p (cell wall protein)	48.2 kDa	5.8
YJL171C	Toh1p (cell wall protein)	42.9 kDa	18
YGR282C	Bgl2p (cell wall protein)	34.1 kDa	2.6
YNL055C	Por1p (mitochondrial porin)	30.3 kDa	37

### Transport coupling efficiencies of Pma1p in its basal and activated states

**Figure 2:**
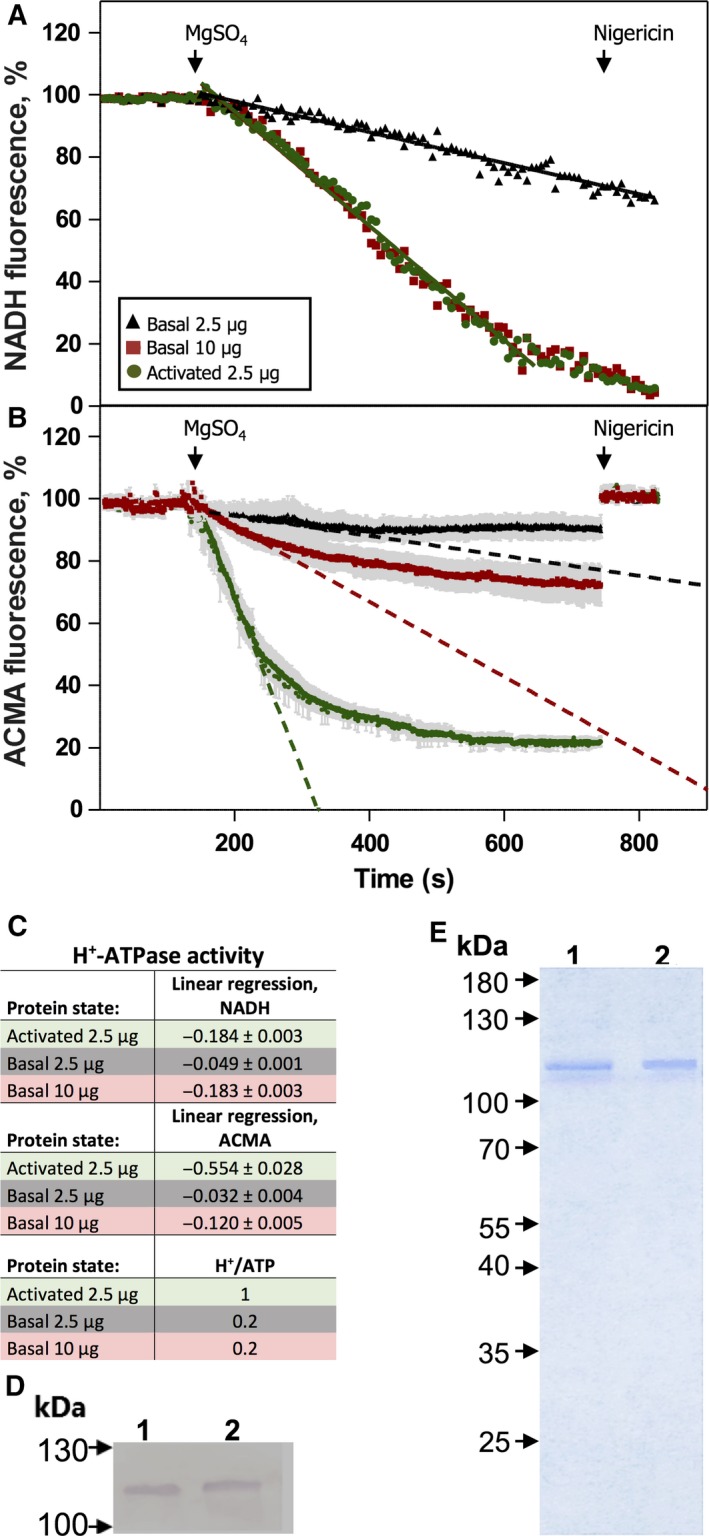
Proton/ATP coupling ratio is not affected by purification. Purified Pma1p in both activation states was reconstituted into lecithin vesicles and the (H^+^ transport/ATP hydrolysis) coupling ratio was analyzed. (A) ATP hydrolysis by Pma1p (2.5 μg) in the activated state (●) is about four times higher than that in the basal state (▲). As a comparison, ATP hydrolysis mediated by four times the amount of protein (10 μg) in the basal state is shown (■). Hydrolysis of ATP was coupled to the reduction in NADH measured as fluorescence quenching at 340 nm. (B) Initial rates of H^+^ pumping by Pma1p (2.5 μg) in the activated state (●) are much higher than in the basal state (▲). As a comparison, H^+^ pumping by four times the amount of protein (10 μg) in the basal state is shown (■). H^+^ transport was detected by monitoring the quenching of ACMA fluorescence. The assay was started after 150 s by adding 8 mm MgSO4 (final concentration), and the pH gradient generated was collapsed after 750 s by adding nigericin. Fluorescence before MgSO4 addition was set to 100% and the difference in coupling ratio was estimated using linear regression in the linear area of the ACMA fluorescence curve. (C) The H^+^ transport/ATP hydrolysis coupling ratio is approximately five times higher in the activated state than in the basal state, that is, H^+^ pumping efficiency per ATP split increases fivefold when the pump is post‐translationally activated. The maximal number of H^+^ per ATP hydrolyzed in the activated state is one 11. With a given value of one H^+^ per hydrolyzed ATP for Pma1p in the activated state, the number of H^+^ per hydrolyzed ATP in the basal state is calculated based on the difference between the linear regressions of fluorescence. (D) Immunoblot of purified Pma1p (2.5 μg) in the activated (lane 1) and basal (lane 2) state as a control for equal amount of Pma1p protein in the assay. (E) Coomassie Blue stained SDS/PAGE gel (10%)‐purified Pma1p (2.5 μg) in the activated (lane 1) and basal (lane 2) state as a control for the same purity of the two preparations.

We previously showed that ATP hydrolysis is partly uncoupled from H^+^ transport in Pma1p, when in its basal state 21. However, it is not known whether this is a stable intrinsic property of the polypeptide. To test whether the purification procedure affected the coupling ratio of Pma1p, we measured proton pumping into Pma1p‐embedded liposomes using the fluorescent probe ACMA. Simultaneously with measuring proton transport, we detected the ATPase activities in the same well by coupling ATP hydrolysis to NADH fluorescence using phosphoenolpyruvate, pyruvate kinase, and lactate dehydrogenase, which convert the produced pyruvate into lactate by oxidizing NADH to NAD^+^. The ATP hydrolytic activity was 3.7 times higher in the activated state (Fig. 2A,C), whereas H^+^ transport was 17.3 times higher in this state than in the basal state (Fig. 2B,C). The purity of Pma1p was equal in both activation states as determined by Coomassie Blue staining of SDS/PAGE gels and immunoblotting using an anti‐Pma1p antibody (Fig. 2D,E). Assuming that one H^+^ is transported per ATP hydrolyzed in the activated state 14, the apparent coupling ratio of the basal state was around 0.2 H^+^ pumped per ATP hydrolyzed. When protein concentrations were adjusted between the two states to give the same ATP hydrolytic activity, similar results were obtained (Fig. 2A–C). The exact coupling ratio cannot be determined with certainty as we do not know whether all Pma1p molecules become activated following the addition of glucose 10. Furthermore, it is possible that part of the H^+^ pumping activity observed for Pma1p in the basal state is due to a fraction being in the activated state 14, 21.

### Pma1p stability

**Figure 3:**
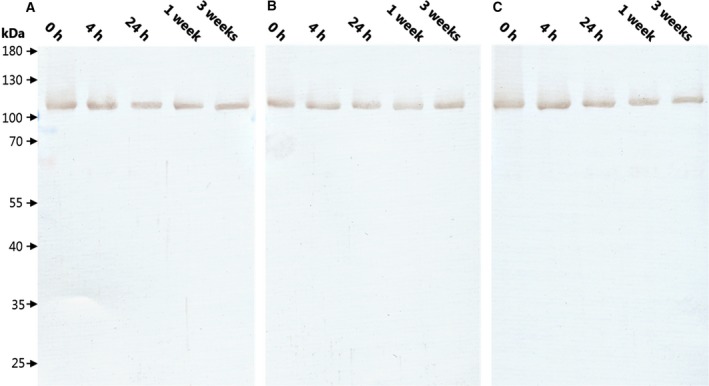
Long‐term stability of purified Pma1p. Purified Pma1p in the basal state was incubated with either (A) no inhibitor, (B) 1 mm AlFx, or (C) 1 mm ADP‐AlFx for up to 3 weeks. Samples of 1 μg protein were precipitated with trichloroacetic acid at the given time points, and their polypeptide composition was analyzed by immunoblotting using anti‐Pma1p antibody.

To test the stability of the Pma1p preparation, we incubated the purified protein for up to 3 weeks at 4 °C and analyzed the degradation using immunoblot with anti‐Pma1p antibody. As phosphate analogues such as aluminum fluoride (AlF_x_) and beryllium fluoride (BeF_x_) inhibit Pma1p 21, we incubated the Pma1p in the basal state with either AlF_x_, which locks the protein in the E2P conformation, or with ADP‐AlF_x_, which locks Pma1p in the E1P conformation. The protein was stable for at least 3 weeks both with and without inhibitors, and no degradation products were identified (Fig. 3).

## Discussion

### Stabilization of Pma1p in the basal state does not require accessory proteins

Stabilization of the autoinhibited state of PM H^+^‐ATPase may require association with other proteins. Indeed, acetylated tubulin has been shown to interact with Pma1p in the basal state only and the addition of acetylated tubulin can even inactivate Pma1p *in vitro* 22, 23. We therefore expected that acetylated tubulin would co‐purify with Pma1p in its basal state. The absence of acetylated tubulin from the purified autoinhibited Pma1p protein demonstrates that acetylated tubulin is not essential for keeping Pma1p in the basal state. Alternatively, acetylated tubulin may be involved in rearranging or mediating the oligomerization of Pma1p in the membrane. A number of contaminating proteins were still present in the purified Pma1p preparation but in very low amounts to compared to Pma1p. Interaction with an external protein partner can therefore not be a requirement for keeping Pma1p in the basal state.

### Regulation of transport coupling ratios is an intrinsic property of the PM H^+^‐ATPase

In this study, we have shown that the yeast H^+^‐ATPase can be purified without the use of affinity tags and that both activation states of the protein can be stably maintained using this protocol. This allows, for the first time, for the kinetic properties of both regulatory states of the purified PM H^+^‐ATPase to be compared directly. In a previous study employing reconstituted plasma membrane vesicles and not purified protein, it was suggested that the basal state of Pma1p has a low coupling ratio between ATP hydrolysis and protons pumped, whereas the glucose‐activated state has a high coupling ratio 14. Using the purified native preparations reported here, we were able to confirm this finding and demonstrate that activation of the PM H^+^‐ATPase causes an approximately fivefold increase in its H^+^ pumping efficiency.

Recently, the kinetics of the plant PM H^+^‐ATPase AHA2 at single molecule level were analyzed, and the transport properties of the wild‐type and a C‐terminally truncated pump were compared 32. However, as both the wild‐type and mutant proteins were tagged, and even carried different tags attached to different parts of the molecule, which may interfere with its catalytic properties 32, the results are hard to interpret. The method presented in this work allows for direct comparison of the properties of native PM H^+^‐ATPase in both activation states and could be useful for future single molecule measurements.

### Perspectives for future structural studies

Progress in our understanding of the structure of fungal PM H^+^‐ATPases has been sparse since 1998, when a 2D structure at 8 Å resolution was published 30. Although several factors may have contributed to this slow rate of progress, difficulties in obtaining defined conformational states are likely to have been among these. Several other members of the P‐type ATPase family have been crystallized 4, and at present, more than 100 X‐ray crystal structures are available in the Protein Data Bank. All crystallized P‐type ATPases have been stabilized with inhibitors shown to lock the P‐type ATPases in a defined conformation of the catalytic cycle. The metal fluorides magnesium fluoride, beryllium fluoride, and aluminum fluoride function as phosphate analogues and have been useful in crystal studies, especially of the SERCA Ca^2+^‐ATPase 46, 47. As these fluorides also inhibit Pma1p in both activation states 21, they may prove useful for the crystallization of the pump protein purified in a defined regulatory state as described here.

The fungal PM H^+^‐ATPase has been heralded as a novel target for fungicides; however, only a few potent inhibitors have been reported to date and all of these inhibit other P‐type ATPases too [e.g., refs. 48, 49, 50]. A high‐resolution crystal structure will not only provide more detailed knowledge of the structure and autoinhibitory function of fungal PM H^+^‐ATPase, but will also be useful in the search for potent and specific inhibitors of this essential fungal enzyme. The Pma1p purification protocol described here brings us a step closer to determining the high‐resolution structure of the fungal PM H^+^‐ATPase in both activation states.

## Author contributions

JTP and MP planned the study and wrote the first draft of the manuscript. JTP performed all experiments except for the LC‐MS/MS measurements. TK and GD performed the LC‐MS/MS measurements. All authors took part in the writing the final version of the manuscript.

## Supporting information


**Fig. S1.** Cells were harvested during stationary growth of YAK2 cells.

**Table S1.** Full list of LC‐MS/MS identified proteins in samples with isolated Pma1p.
