# Supplementary figures and images for: Isolation of native plasma membrane H+‐ATPase (Pma1p) in both the active and basal activation states
Source: FEBS Open Bio. 2018 Mar 25;8(5):774–83. doi: 10.1002/2211-5463.12413 (PMC5929935; doi:10.1002/2211-5463.12413)

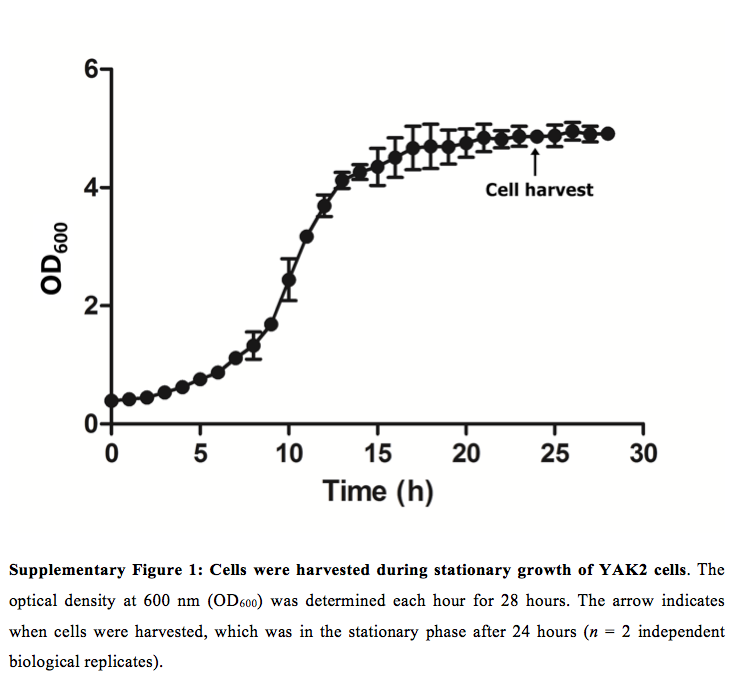

Supplement: Supplementary file 1 — Fig. S1. Cells were harvested during stationary growth of YAK2 cells. [file FEB4-8-774-s001.tiff]
